# Importance of evaluating health-related quality of life issues in survivors of WHO grade II or III gliomas

**DOI:** 10.1093/nop/npab067

**Published:** 2022-01-25

**Authors:** Linda Dirven

**Affiliations:** Leiden University Medical Center, Department of Neurology, Leiden, The Netherlands; Haaglanden Medical Center, Department of Neurology, The Hague, The Netherlands

Gliomas account for 80% of all malignant primary brain and CNS tumors in adults.^1^ About 45% of these glial tumors are composed of several different histologies, including grade II and III astrocytoma and oligodendroglioma. Patients with WHO grade II/III glioma are relatively young at diagnosis, with a median age ranging between 35-56 years depending on the histological and molecular subtype.^2^ The prognosis of grade II/III glioma patients is favorable compared to grade IV glioblastoma, with a median overall survival ranging between only 2 years for grade III astrocytoma IDH-wildtype tumors to more than 17 years for patients with a grade II oligodendroglioma IDH-mutant and 1p19q codeleted tumor.^2^ All glioma patients will be considered to undergo maximal safe resection, but adjuvant treatment regimens differs between subgroups. Whereas low-risk WHO grade II patients (i.e., age < 40 years, gross total resection, preoperative diameter ≤ 6 cm, and well-controlled seizures) will undergo observation only, other subgroups typically receive radiotherapy and/or chemotherapy.^2^ Both the tumor and its treatment impact not only survival but also the patients’ functioning and well-being. Studies that have evaluated the impact of the disease and its treatment on the patients’ functioning and well-being, typically with patient-reported outcomes (PROs) such as health-related quality of life (HRQoL) questionnaires, have focused mainly on the short-term impact. Consequently, less is known about long-term experiences in grade II/III glioma survivors.

In this issue of *Neuro-Oncology Practice*, Frances and colleagues^3^ provide an overview of HRQoL issues in survivors of grade II/III glioma patients, which ultimately can be used to tailor decision-making regarding patient support and treatment. By means of a well-conducted systematic review, 21 quantitative or mixed-methods studies were identified that report on HRQoL issues in grade II/III glioma patients at least two years after diagnosis. Patients reported a wide variety of issues, with negative emotional, psychological, and cognitive changes prevailing. In addition, symptoms such as fatigue and seizures were reported as were limitations in activities of daily life and social relationships as well as financial problems.

With only a limited number of studies available on longer-term issues in grade II/III glioma survivors,^3^ it remains difficult to inform clinical decision-making. This is further hampered by the heterogeneity of the identified studies in terms of study population, design, and selected outcome measures. Indeed, many identified studies in this systematic review^3^ reported on mixed glioma or primary brain tumor populations, thereby impacting the study findings, and only a few studies reported the molecular status of patients. To properly inform clinical decision-making in individual patients, it is important to collect information on the patients’ functioning and well-being in the different histological or molecular subgroups that warrant different treatments. For example, the longer-term HRQoL issues for low-risk WHO grade II patients who undergo MRI surveillance only will be different from patients with higher-risk tumors for whom early radiotherapy and/or chemotherapy is initiated. Also, the time since diagnosis should be taken into account. While it is important for patients and physicians to have insight in the immediate treatment-related toxicity effects, information on the mid-term as well as the (very) long-term HRQoL issues is also needed, as problems and symptoms may change over time. Whereas physical symptoms are predominant in the (pre)diagnostic^4^ and early phase of the disease, issues with emotional, cognitive, and social functioning are reported more commonly in the period after completion of first-line anti-tumor treatment.^3^ In addition, late toxicity effects of the anti-tumor treatment may also occur in the very long-term period, such as radiation necrosis,^5^ which may subsequently have a negative impact on the patients’ (neurocognitive) functioning and well-being. In the current review,^3^ the definition of long-term survivor (i.e., mean or median time since diagnosis of ≥ 2 years) unintentionally resulted in the inclusion of patients who were assessed < 2 years after their diagnosis, hampering interpretation of long-term HRQoL issues. This limitation is inherent to the cross-sectional study design of many identified studies. These observations emphasize the need for prospective longitudinal studies in well-defined subgroups of glioma patients to better capture relevant HRQoL issues over time.

This brings us to the next limitation, which is the heterogeneity in outcome measures used to assess aspects of HRQoL.^3^ This is emphasized by a recent systematic review showing that 215 different multi-item or single-item PRO measures were used in brain tumor studies to assess aspects of functioning and well-being.^6^ Currently, it is not clear which PRO measures are most relevant for brain tumor patients. In addition, it may be questioned whether the existing static questionnaires, consisting of a fixed set of items, cover all the aspects that are relevant for a specific group of patients. For example, those issues that are relevant for long-term survivors, such as problems with career opportunities, obtaining loans or insurance policies, or relationships with family and friends, may not be sufficiently covered. One solution may be to use item libraries,^7,8^ i.e., large databases with multi-item and single-item scales, that can be used to create questionnaires that are optimized for the setting and population under investigation.

Lastly, the current systematic review^3^ focused on patient-reported outcomes only. Although it is important to gain direct insight in the patient experience, more objective outcomes such as neurocognitive functioning may provide additional insights on the impact of the treatments in the longer term and should be considered in future prospective studies.

In conclusion, the systematic review conducted by Frances and colleagues^3^ provides a highly relevant overview of the current evidence around long-term HRQoL issues in grade II/III glioma patients. The identified caveats may guide future research with the aim to gain better insight in the level of functioning and well-being of patients on the longer term. This information may subsequently be used in clinical decision-making to identify patients in need of support. In addition, this information could facilitate the development of interventions aimed at improving aspects of HRQoL. The ultimate goal is to optimize the quality of survival of this rare patient population.
